# The Transactions of the American Medical Association

**Published:** 1852-05

**Authors:** 


### REVIEWS.

ART. V.—The Transactions of the American Medical Association, instituted 1847; Vol. IV; 8vo. p. p. 677.' Philadelphia, 1851, with four colored plates and a map.

The Transactions of the American Medical Association as they appear, year by year, commend themselves to the public, and particularly to the medical profession, by their freedom from all parade; by the clear and evident purpose, running through every part of them, to advance the profession in every thing that is useful and honorable, and to subserve the interests of the community at large by the promotion of well-considered knowledge both theoretical and practical. We have read the last volume with much pleasure, particularly the report of the Committee on Practical Medicine. The short notices which we propose to present, are not intended so much as a review, or criticism, as something added—the observations and conclusions of another physician collateral to those of the committee.

*Typhoid Fever.*—The committee say of this malady: "The principles that should govern the management of typhoid fever can not be considered to be definitely settled.****As regards the feasibility of arresting the disease at its commencement; of abridging its duration; the choice of different means proposed for these ends; and during the febrile career, the extent to which the practitioner should interpose medical influences, the particular remedies indicated, the employment of stimulants, alimentation, etc. On these various points different views are entertained; and hence the subject is open for continued investigation, and for the accumulation of facts," and, let us add, *for the settlement of some principle.*

To the philosophical thinker it must appear very strange that in a disease so well defined as typhoid fever, there should be so great a diversity of opinion, upon the points stated in the above quotation. What is typhoid fever? According to Louis, Andral, and others in France, and according to Gerhard and Bartlett in the United States, it is a fever, the essential element or pathognomonic sign of which is a peculiar form of inflammation of the bowels; it is a dothein-enterite; a fever which begins with a lesion of certain glands in the small intestines. According to Dr. Bartlett, there are the following forms of fever: typhus, typhoid, periodical, *i. e.* intermittent and remittent fever, and yellow fever; and no more. Every case of fever must belong to one or the other of the above classes. Dr. Bartlett practiced medicine for some five or six years in this city, and he does not pretend to say in his work upon fevers that he ever saw a case of typhus or yellow fever in Kentucky. The fever cases which he treated in and about Lexington, he pronounced to be, all, cases of typhoid fever. The exanthemeta are of course excluded: and Dr. Bartlett may have had some few cases of remittent or intermittent fever; but about the diagnosis of these there could be no mistake. The continued fever of this region of Kentucky he pronounced to be typhoid fever. The question is,—is there not another form of fever which prevails in the United States, and especially in Kentucky, *besides the fevers laid down by Dr. Bartlett?*

Is there not a continued fever, which is neither yellow fever, nor typhus fever, nor periodical fever, nor typhoid fever? We maintain that there is. We maintain that there is a continued fever of a typhoid character, which is not a dothein-entrite; a fever in which there is not the peculiar lesion of the small intestines, which is the essential element of typhoid fever; without which lesion the fever can not exist.

During the autumn of 1850, we treated 16 cases of fever, in and about Lexington, not one of which continued over ten days; that is, they were all fairly convalescent at or before the expiration of ten days. Were these sixteen cases, cases of typhoid fever? We answer that they were not, in our opinion, cases of dothein-enterite; that they were not cases in which there was inflammation of Peyer's and Brunner's Glands. But we feel satisfied that Dr. Bartlett would have pronounced them cases of typhoid fever. According to his definition and classification, what else could they have been? They were cases of fever, of idiopathic fever; and it is positively certain that they were not cases of typhus fever, nor of yellow fever, nor of intermittent or remittent fever. Were they cases of typhoid fever; we mean typhoid fever as defined by Andral, Louis, Gerhard and Bartlett? We maintain that they were not.

If we are asked to describe the disease, we answer that we can not distinguish it from genuine typhoid fever in its early stages. There is soreness of the bowels upon pressure, and there is diarrhea. We have no recollection of ever having seen the rose-colored spots peculiar to typhoid fever.

Indeed they are such cases as are constantly called typhoid fever and treated as such. And hence in our opinion arises the great discrepancy of statement among physicians as to cutting short the disease, as to duration and as to treatment. If these cases are treated with cathartics, with a view to exite the action of the liver, and keep up bilious discharges until the fever is broke, they will very generally terminate in from 8 to 14 days. But if these very cases are put upon the expectant plan, and dosed with opium and astringents, they will continue for 21 days or longer, and there may be induced lesion of the bowels. So that it can be clearly seen that if one physician treats twenty cases of this form of fever upon one plan, and another treats twenty cases of the same form of fever upon another plan, there may be a wide difference as to the time the cases may be on hand; and one may say that he has cut short his cases. We wish to be clearly understood not to deny the existence of typhoid fever as defined by Louis and Bartlett. We have treated many cases of it, and have witnessed many post mortem examinations in which the lesions were perfectly manifest. Nor do we believe that such cases can be cut short. We are writing to show that there is another fever (of a typhoid type) which is not a dothien-enterite; a fever in which there is not inflammation of the bowels; and we maintain that this fever, if properly treated, ought not to continue longer than from 8 to 14 days.

It has been doubted by some, we believe, whether typhoid fever exits in England. Many of the British physicians make no distinction between it and typhus; though it has been proven that the peculiar lesion of typhoid fever is not found, post mortem, in the subjects of typhus fever. The relapsing fever, now the object of particular attention in England, is not typhoid fever, nor is our fever relapsing fever. There is no such lesion in relapsing fever as there is in typhoid fever. The question then arises, have we not a continued fever in the United States and especially in all limestone regions, of a typhoid form or character in which there is no inflammation of the bowels? We believe that there is.

There can be no sense or reason in medical science, if a strong cathartic course of treatment will succeed equally well with an expectant or palliative one, or with a method, the principal means of which are opium and astringents, in a fever, where the peculiar lesion is, a very bad form of inflammation of the bowels. Dr. Bartlett, in his work on fevers, admits that there is no great difference in the percentage of deaths between the cathartic and the expectant modes of treatment. And we know physicians who have the full confidence of their patrons and who are considered very successful in the treatment of typhoid fever, who use cathartics throughout the course of the fever, and that too in large doses; and these physicians practice in the midst of others who rely upon opium and astringents. We may depend upon it, the lesion does not exist in every case. May we not suppose that as civilization advances, as the manner of living becomes more and more artificial, as hereditary cachexia from its various sources is spread more and more widely among the people, there will be a tendency to localization on the bowels in continued fever; but that whether this man or that will be so affected, will depend upon his own constitution?

The conclusions, then, at which we have arrived, are these: 1st. That the reason why there appear to be so remarkable differences among physicians upon the points stated at the head of this article, is because the cases are not all typhoid fever in the sense of Louis and Bartlett.

2nd. That the cathartic treatment will prevent all such cases from being protracted; whereas, under the expectant or opium plan they will be very likely to become so.

A few words, in conclusion, as to our plan of treatment. We are very careful to keep the skin of the patient clean; and to insure this, we generally have them washed all over at the commencement of the attack. If the fever is high, and the skin very hot when we are called in, we advise cold water; if otherwise tepid or warm water. We rely very much upon bathing and sponging to keep down the fever throughout the case. If there is diarrhea, we use calomel and Dover's powder—as a general thing, gr. v. áá. at a dose, to be repeated every 4, 6, or 8 hours, according to circumstances. We continue this course with variations until the patient is convalescent. We use also rhubarb, aloes, and compound extract of colocynth; always such cathartics as are known to act on the liver, and we carefully avoid such cathartics as have a tendency to excite serous discharges. Our opinion is, that the reason why cathartics have been so much found fault with by some is, because such cathartics have been used as excite serous discharges. We do not object to astringents; and often use them, but our main object is to keep up a gentle action on the liver, and this we endeavor to effect by the mildest means possible. When in any case we become satisfied that the peculiar lesion of typhoid fever exists, we are very careful about giving a cathartic, and then pursue a palliative treatment.

We are satisfied that patients treated on the cathartic plan even in typhoid fever proper, in its early stages, recover much more rapidly than where cathartics are excluded; and that there is not near so much danger of the common sequelæ;—such as lame limbs, dropsies, bad coughs, scrofula, &c., &c.

One word concerning quinine:—we know of no form of fever in this climate, in which quinine is of any value except intermittent and remittent fever.

*Ague and Fever.*—It may be thought that a physician living at Lexington, Ky., where there hardly occurs one case of ague and fever in five years, is not very well qualified to write about it. But we have this to say:—during our childhood and boyhood we had this hateful malady for nine successive summers; so that we may claim to have a personal knowledge of its symptoms, and to be well acquainted with its odious character. The first correct knowledge we got of its treatment was in Huntsville,

Alabama, near which town we lived for two years and commenced the study of medicine. We have since had some reputation for "curing" it; and treat perhaps as many cases every year as any physician who does not live in an agne and fever district (where we never intend to live again if we can help it.) Nearly all the cases which we have treated originated out of the state. They are persons who have been traveling in Indiana, Illinois, and Missouri. Many of them have had the disease for a year; some for two years or longer, and all have taken quinine. Many say that they will take no more quinine, as it injured them; that it would stop the chills, but not prevent their return; that they have come to us for a permanent cure, though some say that they have despaired of ever being cured. It is a rare thing for them to be disappointed. We have yet to learn the first case which was not cured; and it is very seldom that a patient has the second chill after commencing to take our medicines. We have given our formularies to many physicians, who practice, some in Alabama, some in Missouri, and other states where the disease prevails; and they write us that our pills are in great demand: that they succeed better than any other remedy. The composition of our pills is to the patient a secret, so that they do not know what they are taking. We always tell every patient that we will certainly cure him so that we have the full benefit of his faith. We give two boxes of pills; "ague pills," and "cathartic pills." The latter are composed as follows: R. Calomel, Sulphate of Iron, Socotrine Aloes, áá ℈ij.m.ft. xxiv pills; after reducing the sulphate of iron and aloes to an impalpable powder and mixing all together, we add whisky to make the pill mass; it is better than gum, as the pills more readily dissolve.

For the ague pills we have this formulary: R. Sulph. quinine, Dovers powder, áá ʒj., Cayenne pepper, Soc. aloes, áá ʒss.: mix with whisky and make 36 pills.

We direct the patient to commence taking the ague pills at least 8 hours before the time for the chill or ague and to take a pill every hour until he has taken six pills. He will have taken the last pill two hours before the paroxysm. If he can begin twelve hours before the paroxysm and take a pill every two hours until he takes six pills, it is still better. We consider it very important that quinine should not be given nearer than two hours to the time of the paroxysm. For if the doses previously given will not prevent the paroxysm on that day, it will only aggravate the case to give it just before the time. If a physician is called to see a patient just before an ague, or when the patient is beginning to get cold, and desires to lessen its severity and to prevent the subsequent fever, he must give his patient a full dose of laudanum. This will in a great majority of cases prevent the fever, if it does not very greatly moderate the chill.

On the well day, in third day ague and fever, we direct the patient to commence taking the pills at the same hour, *i.e.* at least 8 hours before the time of paroxysm and to take a pill every two hours until he takes four pills. After the disease is broken we direct him to take three of the ague pills a day, say one just before each meal, till he has taken them all (36). He is to take at the same time the cathartic pills every night or every other night. It is a very rare thing for a patient to become salivated under the use of these medicines; but if the gums become sore or if there is any good reason why calomel should not be given, then we direct the following pill to be given: R. Soc. aloes, Comp. ext. colocynth, Sulphate of iron, áá ℈j.: mix with whisky and make 12 pills.

The dose of these pills is the same as that of the ones containing calomel. They are an excellent pill in all cases where a decided cathartic effect is desired, and are especially adapted to cases of jaundice, chronic disorder of the liver and spleen and such cases as arise in miasmatic countries, not excepting diarrhea. We also use the Prussiate of iron in making an ague pill. The formulary is as follows: ℞. Sulph. quinine, Prussiate of iron, áá ℥j. Cayenne pepper, Soc. aloes; áá ℈j. Sulph. of Morphine gr. ij.: mix with whisky and make 32 pills. This combination will not as uniformly agree with patients as the first; it is more frequently rejected by the stomach, and oftener causes headache; but yet it suits many cases. It is the combination we most commonly give to children, in powder and in small doses. We use the same cathartic pill and follow the same general course as already laid down. These are the facts—a few words of remark: First as to ague and fever countries—we know of no section of country where this malady ever prevailed, where it does not prevail to this day, with this exception: if cities are built up, and the ground covered with houses and pavements, it may be banished; but it can not be otherwise. Clearing up lands, and draining ponds may remove the disease from particular locations, but it will still prevail in the neighborhood, along water courses, and the like. It is the disease of certain geological formations as typhoid fever is of other formations.

2d. The average duration of life is lower among the white population residing in an ague and fever country, than in those sections of country where it does not prevail. This does not apply to the black race. They enjoy better health in miasmatic regions, subject to intermittent fever, than they do elsewhere; they are much less liable to scrofula and cachexia africana. Both of these latter affections are exceedingly rare among the negro population in the tide water region of Virginia, where the ague and fever prevail every summer. In the limestone region of Kentucky the majority of negroes die of some form of glandular affection. But we do not recollect ever having seen a negro with ague and fever in Kentucky.

3d. Quinine by itself is not a reliable remedy in the treatment of intermittent fever. It is the best of all remedies to stop the chills; but it does not in our opinion possess much tonic power. It is an anti-periodic. It does not build up the system, to fortify it against renewed attacks. Quinine given in conjunction with opium is much more agreeable to the patient and is not near so apt to disturb the system. We have remarked that our patients tell us that they can't take quinine, and yet they take pills without complaint. It is true that they sometimes make a patient very sick, but there are none of the effects of quinine complained of. We consider the quinine, opium, and cayenne pepper to be a happy combination for this malady; but we attribute not less good effect to the iron in combination with the calomel and aloes. Tonic medicines, of which iron may be called the most decided tonic, may be given in combination with cathartic medicines much earlier than is commonly supposed. If physicians will try our pills, they will have nothing to complain of from their tonic properties.

4th. We deem it all important, that in the treatment of intermittent fever, the medicines should be continued for some days after the chills are stopped, and we direct our patients to take all the pills, (32 or 36 ague pills, and 24 cathartic,) before they stop. Of all the sick people that we ever treated, those who live in ague and fever districts and those who have this disease are the most opposed to taking medicine. It is an old saying, that when you ask a man in eastern Virginia, or in Illinois or Missouri how his family are, he will answer that they are all well except that the children or his wife or servants have the ague and fever. They get accustomed to it; they know that it hardly ever kills of itself; they have found that medicines have not much effect upon it, and hence their indifference. This must be overcome if you would cure them radically. There is no disease which undermines the constitution more certainly, and therefore there is none that a greater effort should be made to cure.

We candidly believe that it is in the power of physicians greatly to improve the health of communities residing where this malady prevails.

Where there is not an absolute necessity for it, neither the family nor domestics should go out until after sunrise; nor until they have taken their breakfast, during the months of August and September. We know some members of a family who resided near Louisville when that town was almost a grave-yard, and they made it a rule never to open the doors till nine o'clock in the morning during the sickly season, and they all escaped the prevailing sickness. We had this information from a member of the family. It is also much safer to sleep up stairs, and cold bathing in the morning is a good prophylactic.

Two suggestions more as to the treatment: To prevent a chill when they are obstinate, if you will rub the back up and down the spine with strong and hot red pepper tea, or with dry mustard, half an hour before the time, it will very generally prove efficacious.

There is no form of fever in which the free external application of cold water is a safer and more efficient and pleasant remedy, during the hot stage, than that of intermittent and remittent fever. Both bleeding and emetics do more to prostrate a patient, burning hot with fever, than they do good—often times neither will even stop the fever, and if they do, the patient is left greatly debilitated. But take a patient with a burning skin, a full and excited pulse, and a severe headache, and strip him and lay him down and dash buckets full of fresh cold water over him until the heat of the skin is removed, and then quickly rub him dry and put him to bed, and give him a cup of hot tea, and ten to one he will go to sleep immediately and break out in perspiration. We are in the constant habit of using cold water freely in typhoid as well as intermittent and remittent fever. When the skin is very hot, and especially when there is great heat and disturb ance about the head, we have the patient stripped and the cold water poured over them; but if they are not remarka bly hot, but are only feverish and restless, we then prefer sponging. The feelings of the patient ought to be consulted as to the temperature of the water, where it is only proposed to sponge the patient.

It will be observed that we do not agree with Dr. T. S. Bell as to the use of large doses of quinine in the treatment of this malady. It is sufficient to say, that we have not had occasion to give his method a trial. We believe that those who will try ours will be pleased with it.

*Dysentery.*—Nitrate of silver would seem to be the favorite remedy advocated: it is relied upon and prepared by physicians living in different states and hundreds of miles apart. Its use by injection is the preferred method of administering it; though it is also given in pills. Some physicians rely almost altogether upon opium, though none it would seem have adopted the use of Dovers powder, as recommended by Dr. Watson in his (London) Practice of medicine. Calomel is rather objected to, and when used advised in small doses. Dr. —— is an exception. He gave calomel in 20 and 30 grain doses. Each physician appears to be satisfiied with the result of his experiments and recommends his practice. The opinion is advanced, that the former method of treating dysentery, founded upon the opinion that dysentery is produced by disease or disorder of the liver is erroneous both in theory and in practice.

When it thus appears that dysentery has been successfully treated by the use of three remedies differing so much in their therapeutical effects as nitrate of silver, opium, and calomel, to which number of remedies a long list might be added; it is the part and the duty of the medical philosopher to inquire the reason. Dr. Stone of New Orleans, in his second article on the Phosphate of lime in consumption, &c., (New Orleans Monthly Medical

Register, Vol. I. No. 4,) very properly remarks: "The best way to secure for any remedy its proper place in therapeutics, is to determine its mode of action." This is a piece of old philosophy re-affirmed. It is time that it should be restated by men occupying the station of Dr. Stone; for it is being too much lost sight of. But if such is the best way to secure for any remedy its proper place in the treatment of a particular disease, what are we to say when we find that a severe and painful and even dangerous malady may be cured (so to say) by many remedies? Take the one now before us. We find that physicians claim almost equal success with nitrate of silver, opium and calomel; and we may add, that we have known an extensive epidemic—dysentery—successfully treated as follows:—a teaspoonful of ipecacuanha and a teaspoonful of the sulphate of magnesia were mixed in a pint of water, and a teaspoonful of the mixture was given every hour or every two hours, or seldomer *pro re nata.* Hardly any other medicine was given but this, and nearly every case recovered. We have ourself treated many cases successfully with the following mixture: ℞. Muriate of soda, (common barrel salt) Sulphate of magnesia, áá ʒij., mint water, cider vinegar, áá ℥j., mix and strain: dose, a teaspoonful after every dysenteric operation. Sometimes we add laudanum to the mixture, if the dejections are very frequent. Several physicians of our acquaintance have used this formulary with very gratifying results. But it does not answer in every case, nor will any remedy. Moreover we are satisfied that the hydropathic treatment of dysentery is not without its merits. A physician of our acquaintance, not a hydropath, has tried injections of ice water in a number of severe cases, and he found them of essential service. The cold hip bath is by no means *a remedy without a reason.* How then are all these things to be explained? There is a reason for everything, and the physician who says that it is not philosophical or

5*

proper to undertake to find out the modus operandi of every remedy will never do much to the honor or benefit of the healing art. Perhaps the most rational explanation is, to say with Dr. Forbes that the vast majority of the curable cases would as certainly get well without a remedy as with it. That if we do not interfere too much and interrupt the recuperative efforts of nature, she will bring all right. This is not only possible but probable. But the disease is so painful and apparently so dangerous in its symptoms, that it is not likely that any considerable number of patients will at any one time and place submit themselves to the purely *expectant method* to ascertain the fact.

There must be some other explanation; and this we think lies in the nature of the disease. Much has been said about the part the liver has to play in dysentery. Some deny its being implicated at all; others maintain that it is always at fault. One thing is certain, that the bile ceases to be poured out into the intestines when the dysentery begins, and the ductus communis choledochus remains occluded until the bloody operations begin to stop. Whether the liver is all this time doing its own proper office of secreting the bile from the venous blood, but has not the power to command the ductus choledochus to do its proper duty, may possibly be a matter of dispute between the horns of which dilemma lies the gall bladder. We have never known a case of dysentery to begin to improve until bilious evacuations appeared; and by the time the disease is removed, the fæces contain their usual quantity of bile. Medical writers upon the practice seem to forget the office of the liver; that its action in the economy is constant and not occasional, and to forget what that office is. When the arterial blood has passed the round of the circulation, almost after having been first oxygenated in the lungs, it makes its final tarry in the liver, where the refuse and debris of the body which have come down into the general current by many little rivulets, have to be separated. This is the first office of the liver. Upon the performance of this office by the liver, the purity and healthfulness of the blood itself depends. Many pathological difficulties affecting different organs arise when this office is not performed. But this is not all by half. This effete matter, this same thing to be removed from the venous blood by the liver is the thing, if separated, which has to be converted into bile. The effete matter may be to some extent removed by the liver, and yet healthy bile may not be made. Here is a double difficulty. And, again, if bile is made, it may not be poured out in a continued stream, as it is in health, into the duodenum. It may stop in the gall bladder or in the ductus choledochus. So that in either contingency healthy bile is not continually poured out into the bowels; indeed, in a large majority of cases, is not poured out at all during the continuance of dysentery. This is *the fact.* But is the liver diseased? You do not find, we are told, the liver diseased as a general fact in the bodies of persons who died of dysentery. We answer that we did not, according to our theory, expect to find organic disease of the liver. A man who has a diseased liver may have dysentery, and persons who have resided in hot climates until their livers become organically affected, are very subject to dysentery. But it does not follow that a man must have a diseased liver before he can have dysentery. A man may be troubled with indigestion or dyspepsia and yet his stomach may not be diseased organically, nor the seat of the malady. This is often the case. But the stomach does not perform its functions notwithstanding. The stomach is often the only complaining organ when the kidneys are diseased; and so of the uterus; and so also of some forms of brain affection. It does not perform its functions in a single instance, and yet it is not the subject of organic disease.

Dysentery arises from various causes, and is made up in different epidemics and in different individuals of several pathological states, which may not be the same in two epidemics, nor in three out of five cases in the same epidemic. Sometimes we have an epidemic dysentery which arises from miasmatic csuses, in which there is manifestly disorder of the liver, evidenced by the appearance of the tongue, pain and fullness in the region of the liver and symptoms of jaundice. Sometimes the disease seems to arise from an unknown cause, like typhoid fever, or scarlet fever ; it attacks persons who are the most healthful and robust. We may refer the origin of a case to improper diet ; but perhaps the patient is the only individual in the household, all living upon the same food, who is attacked. As to the symptoms in such cases we do not find any sign of disorder of the liver, except the fact that bile is not poured out into the intestines. The first thing we know is inflammation of the lower bowels, with bloody discharges and scanty, high colored urine. Now should these two varieties of the disease be treated in the same manner? By no means. In the first instance, ipecac. emetics, or ipecac. and calomel in full doses, with the assistance of the saline mixture; or if the epidemic be a mild one, the solution of ipecac. and Epsom salts will be the best remedies. This is the form in which scruple and half drachm doses of calomel administered every 6, 8, or 12 hours, according to the case, until healthy bilious actions are produced, do so much good. The affection of the liver here is a part of the malady, and the treatment has to be mainly directed to it. But this treatment would not suit the other variety. The liver has merely stopped acting. The severe inflammation of the lower bowel extended in its effects along the track of the mucous membrane to the liver, affects it in the same manner, except that it is more direct, as disease of the kidney or uterus affects the stomach, and simply causes it to stop acting. Remove the cause of irritation, the cause of this extended sympathy, and the organ, whether stomach or liver, will immediately commence anew to do its duty. This is the form of dysentery in which calomel or blue mass is a very questionable remedy. It must be decided for each case whether mercury should be used or not; it can not be decided for an epidemic. This is the form in which we should expect to find a remedy in blood-letting, in the nitrate of silver, in opium, in cold water, and in the saline mixture. A word about this saline mixture, of which we have given the formulary. It is a very curious compound in its effects, considering its composition; not one of its ingredients has ever been considered a remedy for dysentery or diarrhea, given by itself; and in a majority of cases the salt, the vinegar, and the Epsom salts would no doubt do harm if given in the quantities and as often as they are given in the mixture. But yet the mixture, even without laudanum or morphine, (we generally add one or the other where the case is a severe one), will relieve the violent tenesmus, check the bloody and mucous discharges and cause the liver to act, sometimes as if by magic. We commonly give it in conjunction with other remedies; giving them at longer intervals and the mixture after every unfavorable operation, in mild cases; and when the disease begins to subside, we give the mixture at intervals of three or four hours, or only three times a day, and give nothing else.

Perhaps there are no two diseases about which there is less dispute as to the actual lesion than dysentery and pneumonia: and we may, we believe, add that there is a very general concurrence among physicians as to the remedies, and yet we know of no two diseases where the physician should be more careful to examine every case for itself, to see if the treatment must not be varied to suit the case. Tartar emetic will not do in all cases of pneumonia, nor will calomel nor any other drug do in all cases of dysentery—but the nature of the disease is not changed for that reason—as remarked by Dr. Stone, when we have "determined the mode of action of any remedy" we have secured its proper place in therapeutics. But it is not only important to determine the mode of action of a remedy, but also to find out, at any rates, the *proximate* cause of a disease and to trace link by link the chain of morbid causes and effects, and to ascertain whether the final link is rivited into some organ or only tied to it by a slender thread.

In dysentery, arising from miasmatic origin, we would say that the one end of the chain was rivited into the liver and the other into the mucous membrane of the bowels; in the other variety, we would say that the link next to the liver is only attached to that organ by a slender thread.

In the one variety the disease may be said to begin in the liver and end in the bowels. In the other, it begins in the mucous membrane of the bowels and terminates at the liver. In the one case you must arouse the liver and put it in action to arrest the dysentery: in the other you must subdue or assuage the inflammation of the bowels before the liver will act. So far as practicable this distinction should be kept in view: for there is no method more uncertain and more dangerous than the eclectic, when it is made to mean to give a remedy or a supposed remedy for every symptom that arises—a mixed treatment is, of all sorts of practice, the worst. General principles must be always kept in view.

We have not attempted to lay down in the above remarks a plan of treatment for dysentery; our object has been to shew that where the malady has been at one place treated successfully by calomel, and in another by nitrate of silver, it does not argue that either calomel or nitrate of silver is the only remedy or the best remedy for all cases; but that there are various forms of the malady.

J. C. D.

Lexington, April, 1852.

